# Colonización nasal por *Staphylococcus aureus* resistente a la meticilina en pacientes sometidos a cirugía cardiovascular en un hospital universitario de Bogotá, Colombia

**DOI:** 10.7705/biomedica.4791

**Published:** 2020-08-20

**Authors:** Heidy C. Martínez-Díaz, Sandra L. Valderrama-Beltrán, Ana C. Hernández, Silvia K. Pinedo, Juan R. Correa, Édgar G. Ríos, Julie J. Rojas, Yessica Y. Hernández, Marylin Hidalgo

**Affiliations:** 1 Grupo de Enfermedades Infecciosas, Departamento de Microbiología, Facultad de Ciencias, Pontificia Universidad Javeriana, Bogotá, D.C., Colombia Pontificia Universidad Javeriana Departamento de Microbiología Facultad de Ciencias Pontificia Universidad Javeriana BogotáD.C Colombia; 2 Grupo de Investigación en Enfermedades Infecciosas, Hospital Universitario San Ignacio, Facultad de Medicina, Pontificia Universidad Javeriana, Bogotá, D.C., Colombia Pontificia Universidad Javeriana Facultad de Medicina Pontificia Universidad Javeriana BogotáD.C Colombia; 3 Unidad de Cirugía Cardiovascular, Hospital Universitario San Ignacio, Bogotá, D.C., Colombia Hospital Universitario San Ignacio BogotáD.C Colombia; 4 Maestría en Ciencias - Microbiología, Universidad Nacional de Colombia, Bogotá, D.C., Colombia Universidad Nacional de Colombia Universidad Nacional de Colombia BogotáD.C Colombia

**Keywords:** *Staphylococcus aureus* resistente a meticilina, portador sano, mucosa nasal, infección de la herida quirúrgica, mupirocina, infección hospitalaria, Methicillin-resistant *Staphylococcus aureus*, carrier state, nasal mucosa, surgical wound infection, mupirocin, cross infection

## Abstract

**Introducción.:**

*Staphylococcus aureus* resistente a la meticilina (SARM) es un microorganismo que coloniza las fosas nasales y diferentes partes del cuerpo, lo cual se considera un factor de riesgo para adquirir infecciones invasivas, especialmente en pacientes sometidos a cirugía cardiovascular.

**Objetivo.:**

Determinar la colonización nasal por SARM y establecer las características clínicas en pacientes programados para cirugía cardiovascular.

**Materiales y métodos.:**

Se hizo un estudio descriptivo entre febrero y diciembre de 2015. Se incluyeron pacientes adultos programados para cirugía cardiovascular en el Hospital Universitario San Ignacio de Bogotá. La colonización se identificó mediante reacción en cadena de la polimerasa (*Polymerase Chain Reaction*, PCR) en tiempo real en muestras obtenidas mediante hisopados nasales. Los pacientes fueron descolonizados con mupirocina al 2,0 % intranasal dos veces al día y baños con gluconato de clorhexidina al 4 % del cuello hacía abajo durante cinco días, al cabo de lo cual se hizo una PCR de control.

**Resultados.:**

Se incluyeron 141 pacientes, 52 hospitalizados y 89 ambulatorios. Del total, 19 (13,4 %) tenían colonización nasal por SARM, correspondientes a 9 (17,3 %) de los 52 hospitalizados y 10 (11,2 %) de los 89 ambulatorios. Todos los pacientes sometidos a descolonización tuvieron resultado negativo en la PCR al final del proceso y ninguno presentó infección del sitio operatorio por *S. aureus*.

**Conclusiones.:**

Se demostró colonización nasal por SARM tanto en los pacientes hospitalizados como en los ambulatorios. La descolonización con mupirocina fue efectiva para erradicar el estado de portador a corto plazo, lo que podría tener efecto en las tasas de infección del sitio operatorio en las cirugías cardiovasculares.

## Abstract (en)

**Introduction:**

Methicillin-resistant *Staphylococcus aureus* (MRSA) is a microorganism that colonizes nostrils and different parts of the body, which is considered a risk factor to acquire invasive infections, especially in cardiovascular surgery patients.

**Objective:**

To determine the frequency of nasal colonization by MRSA and to establish the clinical characteristics in patients scheduled for cardiovascular surgery.

**Materials and methods:**

This was a descriptive study conducted between February and December, 2015. We included adult patients scheduled for cardiovascular surgery at the *Hospital Universitario San Ignacio* in Bogotá, Colombia. Colonization was identified by real-time PCR from nasal swabs. Colonized patients were treated with mupirocin 2.0% intranasally twice a day and bathed with chlorhexidine 4% from the neck downwards for five days. At the end of this treatment, PCR control was carried out.

**Results:**

We included 141 patients with a percentage of nasal colonization of 13.4% (19/141). There were 52 hospitalized patients and 89 outpatients with a percentage of nasal colonization of 17.3% (9/52), and 11.2% (10/89), respectively. All colonized patients who received treatment had a negative PCR at the end of the regime and none of the participating patients had a surgical site infection by *S. aureus* at the end of the study.

**Conclusions:**

Nasal colonization was observed both in hospitalized patients and outpatients. Decolonization treatment with mupirocin was effective to eradicate the carrier state in the short term, which could impact the rates of surgical wound infection associated with cardiovascular surgery.

*Staphylococcus aureus* es un microorganismo que puede colonizar diferentes partes del cuerpo como la nariz, la faringe, las ingles, la región perianal y las axilas, lo que se considera un factor predisponente para las infecciones invasivas ^1^. Entre el 15 y el 30 % de los adultos sanos presentan colonización nasal por *S. aureus* sensible a la meticilina (SAMS), y entre el 1 y el 3 % por *S. aureus* resistente a la meticilina (SAMR) ^1^^-^^3^. Este ha emergido durante los últimos años y se ha convertido en un microorganismo de gran importancia en salud pública, debido a sus perfiles de resistencia y factores de virulencia ^4^.

Estos dos tipos de *S. aureus* causan infecciones en pacientes con diversas condiciones de salud y en los sometidos a cirugía ^4^^-^^8^. Las infecciones del sitio operatorio en pacientes sometidos a cirugía cardiaca, se asocian con significativas tasas de morbilidad y mortalidad que varían según las condiciones del paciente, el tipo de procedimiento quirúrgico realizado y el microorganismo implicado ^9^^,^^10^. SAMS y SAMR son responsables de más de la mitad de dichas infecciones en casos de cirugía cardiaca, siendo los pacientes colonizados los que presentan mayores probabilidades de adquirir infecciones hospitalarias asociadas con *S. aureus*^9^^,^^11^^-^^13^. A nivel mundial, se reportan actualmente diferentes porcentajes de infección del sitio operatorio por *S. aureus* en pacientes de cirugía cardiaca, los cuales varían entre 24 y 60 % en países como Francia, España y Estados Unidos ^9^^,^^13^^,^^14^.

En Colombia, se ha reportado una colonización nasal con *S. aureus* del 34 % en pacientes de cirugía cardiovascular ^15^; en aquellos atendidos en cuidados intensivos, el porcentaje es de 25,8 %, del cual el 7,2 % corresponde a SARM ^16^. Según los resultados del último informe de la vigilancia de la resistencia a los antimicrobianos en infecciones asociadas con la atención en salud, en el 2017, el perfil de resistencia a la oxacilina en las unidades de cuidados intensivos fue de 34 % y, en otras, de 38,4 % ^17^. En el boletín epidemiológico distrital se informa que, en los hospitales en Bogotá en el 2015, el 32,6 % de las infecciones superficiales del sitio operatorio, el 34,7 % de las profundas y el 23,3 % de aquellas en órgano o espacio fueron causadas por SARM ^18^.

Según las estadísticas del Hospital Universitario San Ignacio, en el 2014, el 29,8 % de los aislamientos de *S. aureus* obtenidos en la unidad de cuidados intensivos y el 37,4 % de los obtenidos en otras áreas de atención del hospital, fueron resistentes a la meticilina. En el Servicio de Cirugía Cardiovascular del hospital se intervienen, en promedio, 240 pacientes al año y, según las estadísticas del 2014, la tasa de mortalidad fue del 8 % y la de infección del sitio operatorio del 3 %; *S. aureus* estuvo presente en el 45,5 % de los casos y, de estos, el 18,2 % correspondió a SARM.

Con base en estas estadísticas y los factores ya anotados, y dado que en el país son pocos los estudios sobre este grupo de pacientes, se consideró importante determinar la prevalencia de la colonización nasal y describir las características clínicas de los pacientes atendidos en el Servicio de Cirugía Cardiovascular del Hospital Universitario San Ignacio, con el fin de aportar información local útil como respaldo al diseño de programas de prevención de infecciones por SARM.

## Materiales y métodos

### Pacientes y muestras

Se hizo un estudio descriptivo de los pacientes atendidos en el Servicio de Cirugía Cardiovascular del Hospital Universitario San Ignacio de Bogotá durante el periodo comprendido entre febrero y diciembre del 2015.

Se incluyeron los mayores de 18 años programados para cirugía cardiovascular que participaron voluntariamente. La selección se hizo entre los pacientes hospitalizados y aquellos que acudieron ambulatoriamente al Servicio de Cirugía Cardiovascular. Se incluyeron 141 pacientes que cumplían con los criterios de inclusión y se recolectaron 190 muestras. El 63,1 % (89/141) fueron ambulatorios y el 36,9 % (52/141) estaban hospitalizados.

Después de la programación de la cirugía y la firma del consentimiento informado, a los pacientes ambulatorios se les tomó la primera muestra de hisopado nasal y, entre las 24 y las 48 horas antes de la cirugía, la segunda. A los pacientes hospitalizados, la mayoría sometidos a cirugía de urgencia, se les recolectó una única muestra antes de la intervención.

### Hisopados nasales

Se recolectaron muestras del tabique adyacente al orificio nasal mediante rotación con escobillón estéril con carbón activado BBL CultureSwab Plus™ y, posteriormente, se remitieron las muestras al laboratorio para su respectivo procesamiento.

### Identificación de SARM

Para la identificación del microorganismo en los hisopados nasales, se empleó la técnica de PCR en tiempo real (qPCR) utilizando el estuche comercial LightCycler MRSA Advance™ y el instrumento LightCycler 2.0™ de Roche.

El ADN de las muestras se extrajo mediante lisis mecánica del hisopado nasal usando el kit LightCycler Advanced Lysis™ y el MagNA Lyser Instrument™, según las instrucciones del fabricante. Posteriormente, se amplificó el ADN mediante PCR y la detección se hizo mediante hibridación específica de sondas.

Después de validar cada una de las pruebas, se analizaron los resultados generados automáticamente por el programa del equipo y se consideraron positivas todas las muestras con valor de C_t_ (*threshold cycle*) inferior a 37 y cuya curva de disociación concordara con la estandarizada, esto, con el fin de identificar solo aquellos amplicones específicos de SARM.

### Profilaxis

En todos los pacientes con resultado positivo para SARM, se inició la descolonización perioperatoria con ungüento de mupirocina al 2,0 % en las fosas nasales, dos veces al día, y baños con gluconato de clorhexidina al 4 % del cuello hacia abajo durante cinco días. A todos los pacientes se les hizo seguimiento durante el periodo de la profilaxis: a los hospitalizados, con visitas diarias en el servicio y, a los ambulatorios, con llamadas telefónicas, con el fin de garantizar el cumplimiento de las medidas y detectar reacciones adversas. Cinco días después de finalizada la descolonización, se recolectó una muestra para evaluar la efectividad a corto plazo de la profilaxis.

Además, a todos los pacientes positivos para SARM se les administró 1 gramo de vancomicina como antibiótico profiláctico en lugar de cefazolina, iniciando la infusión dos horas antes de la intervención quirúrgica y finalizándola entre una hora y 30 minutos antes de la incisión; a esta se agregaron 15 mg/kg de amikacina por vía intravenosa treinta minutos antes de la incisión, según el protocolo institucional.

### Análisis estadístico

Los datos recolectados se extrajeron del sistema de información de historias clínicas (SAHI), del programa del laboratorio clínico LabCore y de la base de datos del Servicio de Cirugía Cardiovascular.

Los datos fueron anonimizados y procesados en el programa Excel Microsoft Office. Para el análisis descriptivo, se utilizaron medidas de tendencia central y de dispersión. Las variables continuas se expresaron como media y desviación estándar si seguían una distribución normal, y, como mediana e intervalo intercuartílico (RIC), si no lo hacían. Las variables categóricas se expresaron como porcentajes. Para establecer la asociación entre la condición de paciente hospitalizado y la de ambulatorio con positividad para SARM, se elaboró una tabla de 2 x 2 para calcular la razón de prevalencias y se calculó la prueba de ji al cuadrado.

Este estudio fue avalado por los comités de ética e investigación del Hospital Universitario San Ignacio y de la Facultad de Medicina y Ciencias de la Pontificia Universidad Javeriana.

## Resultados

Del total de pacientes incluidos, el porcentaje de positivos para SARM se estimó en 13,4 % (19/141) (intervalo de confianza, IC, de 95 %: 0,8-20 %; p*≤*2,2e-16), valor estadísticamente significativo para el grupo de pacientes analizados. El 36,9 % (52/141) de los incluidos estaban hospitalizados y el 63,1 % (89/141) correspondía a pacientes ambulatorios. De estos, el 61,7 % (87/141) correspondieron a hombres y, el restante, a mujeres.

La mediana para la edad del total de pacientes incluidos fue de 64 años, con un rango entre 18 y 83 años. En los hospitalizados, la mediana para la edad fue de 66 años, al igual que para los ambulatorios (cuadro 1). El porcentaje de colonizados por SARM fue de 17 % (9/52) (p=2,038e-06) entre los pacientes hospitalizados y de 11 % (10/89) (p=1,874e-14) en los ambulatorios, lo que representa un valor estadísticamente significativo para ambos grupos.

**Cuadro 1:** Caracteristicas de los pacientes colonizados

Característica	Pacientes hospitalizados (n=52)	Pacientes ambulatorios (n=89)
Edad (anos) (mediana)	66 (58-71)	66 (57-73)
Hombres [n (%)]	38 (73)	49 (55)
Positivos SARM (IC_95_%)	0,17 (0,082-0,30); p≤0,05	0,11 (0,055-0,196); p≤0,05
Indice de masa corporal (media)	25,54 ± 4,6	25,61 ± 3,55
Hipertension arterial [n (%)]	43 (82,7)	30 (33,7)
Infarto agudo de miocardio [n (%)]	14 (26,9)	29 (32,6)
Insuficiencia cardiaca congestiva [n (%)]	19 (36,5)	16 (18)
Enfermedad renal cronica [n (%)]	6 (11,5)	8 (9)
Diabetes mellitus [n (%)]	7 (13,5)	11 (12,3)
Enfermedad pulmonar obstructiva cronica [n (%)]	11 (21,15)	5 (5,6)
Estancia hospitalaria (dias)	19 ± 10,5	8 ± 10,6

Entre las comorbilidades más frecuentes, se destacaron la hipertensión arterial sistémica (82,7 % de hospitalizados y 33,7 % de ambulatorios) y el infarto agudo del miocardio (26,9 %, de hospitalizados y 32,6 %, de ambulatorios). La media del índice de masa corporal (IMC) en ambos grupos fue superior a 25, lo que según la OMS se clasifica como sobrepeso 11 (cuadro 1).

En los pacientes positivos para SARM, se observó que las comorbilidades estadísticamente significativas (p≤0,01) fueron la enfermedad renal crónica (2=10 %) (IC_95%_ 0,1-33 %; p*=*0,0007)*,* la diabetes mellitus (3=15 %) (IC_95%_ 0,3-39 %; p=0,004) y la enfermedad pulmonar obstructiva crónica (2=10 %) (IC_95%_ 0,1-33 %; p=0,0007).

Al asociar la condición de paciente hospitalizado y la de ambulatorio con la positividad para SARM, se observó una razón de prevalencias de 0,82, con lo cual se puede inferir que la frecuencia de colonizados entre los hospitalizados fue 0,82 veces mayor que en los ambulatorios. Se estableció, asimismo, un valor de c^2^=1,038, gl=1 y p=0,308, lo que indicó que no hubo diferencias en la frecuencia de colonizados por SARM entre los hospitalizados y los ambulatorios (nivel de significación, 5 %). Se observó que, en ninguna de las muestras de hisopado nasal recolectadas de los pacientes positivos después de la profilaxis, se detectó SARM y solo un paciente hospitalizado presentó una reacción adversa a la clorhexidina.

## Discusión

El porcentaje de colonización nasal de SARM en los pacientes del Servicio de Cirugía Cardiovascular incluidos en el estudio, fue del 13,4 %, valor estadísticamente significativo en la población estudiada. A nivel mundial, se reportan anualmente prevalencias de colonización nasal por SARM en pacientes sanos de hasta 5,3 %, ^8^^,^^12^^-^^15^ en Estados Unidos y Alemania, y en pacientes hospitalizados de hasta 19,7 %, 20 % y 21,1 % en Estados Unidos, Alemania y España, respectivamente ^14^^,^^16^^-^^18^. En Colombia, se han reportado porcentajes de colonización por SARM cercanos a 7,2 % en personas sanas, a 9,1 % en hospitalizados, a 3,5 % en pacientes con HIV y a 11,1 % en aquellos en hemodiálisis ^19^^-^^21^.

En este estudio, se encontró colonización nasal por SARM en 17 % de los pacientes hospitalizados y en 11 % de los ambulatorios. Además, las comorbilidades estadísticamente significativas en los casos positivos, fueron la enfermedad renal crónica, la diabetes mellitus y la enfermedad pulmonar obstructiva crónica. Estos valores son diferentes a los reportados en otros estudios en Colombia, pero cercanos a la media de los reportados en la literatura internacional ^13^^,^^14^^,^^16^^,^^17^^,^^21^^,^^22^.

Al determinar la asociación de frecuencia de la colonización nasal en los pacientes hospitalizados y los ambulatorios, se encontró que dichas condiciones no fueron factores asociados con la presencia de SARM, de lo que se infiere que, independientemente de la condición de hospitalizado o ambulatorio, el paciente puede estar colonizado y, por lo tanto, las estrategias para mitigar su presencia en el ámbito hospitalario deben cubrir a las dos poblaciones por igual, ya que son una fuente importante de propagación de este microorganismo no solo en ambientes hospitalarios sino en la comunidad. A pesar de la mínima diferencia en el número de portadores entre pacientes ambulatorios y hospitalizados, estos últimos presentan la mayor proporción de colonización, lo que se ajusta a la tendencia reportada.

La colonización por SARM está documentada actualmente como un factor de riesgo para adquirir infecciones hospitalarias causadas por este agente patógeno, especialmente en aquellos pacientes con hospitalizaciones prolongadas, los que han sido intervenidos quirúrgicamente ^5^^,^^23^ y, específicamente, los sometidos a cirugía cardiaca, en quienes el mayor riesgo de padecerlas es implícito ^24^.

La flora endógena de los pacientes sometidos a cirugía cardíaca tiene gran impacto en la fisiopatología de las infecciones del sitio operatorio ^9^ y *S. aureus* es un factor de riesgo importante que se detecta en la mayoría de estas infecciones ^25^. La prevalencia, la complejidad y el éxito del tratamiento de las infecciones en el sitio operatorio pueden variar según el tipo de procedimiento quirúrgico, el microorganismo implicado y las condiciones del paciente, entre otras ^9^^,^^10^.

En Australia, se han reportado aislamientos de SARM y SASM en casos de infección del sitio operatorio en 32 y 24 % de pacientes de cirugía cardíaca, respectivamente ^26^, de *S. aureus* en el 40 a 60 % de las infecciones del sitio operatorio en Francia ^9^^,^^24^, y en el 25 a 35 % de las reportadas en Estados Unidos ^24^.

Según los datos publicados por el sistema de vigilancia distrital, en el 2014 en Bogotá se registró infección del sitio operatorio en 0,11 % de las cirugías ambulatorias y en el 0,09 % de las cirugías que requirieron hospitalización, en tanto que en el 2015 se registró en 0,99 % de las ambulatorias y en 0,91 % de las hospitalarias. SARM estuvo implicado en el 32,6 % de las infecciones superficiales del sitio operatorio, en 34,7 % de las infecciones profundas y en 23,3 % de las de órgano o espacio ^27^.

Para mitigar la propagación de este tipo de infecciones a nivel hospitalario, se han reportado diferentes estrategias de prevención, entre las cuales se encuentra la descolonización nasal con mupirocina acompañada de baños corporales con gluconato de clorhexidina y la profilaxis antibiótica con glucopéptidos, con lo cual se han logrado reducciones significativas de las infecciones del sitio operatorio en pacientes de cirugía cardiaca ^26^^,^^28^^-^^32^.

El análisis de las estadísticas del presente estudio permitió observar una disminución del número de infecciones del sitio operatorio ocasionadas por SARM: en el 2014, el 3 % de los pacientes intervenidos presentaron infección y, de este porcentaje, el 45,5 % se asoció con *S. aureus,* con 18,2 % resistente a la meticilina. En el 2015, año de este estudio, a pesar de que tales infecciones permanecieron en el 3 %, las asociada s con *S. aureus* disminuyeron al 14 %, correspondiente a la infección de un paciente que no hizo parte del estudio por no cumplir con los criterios de inclusión. Cabe resaltar que todos los pacientes a los cuales se les suministró el tratamiento profiláctico dieron resultados negativos después de terminarlo. Sin embargo, se ha demostrado que la mupirocina es efectiva para la descolonización nasal de *S. aureus* durante pocas semanas y la probabilidad de recolonización después de tres meses es alta ^3^^,^^33^, lo que resalta la importancia de hacer seguimiento a los pacientes sometidos a la profilaxis por un periodo largo y de explorar nuevas medidas efectivas de descolonización que garanticen la eficacia a largo plazo.

Por último, según los reportes de la literatura especializada, puede inferirse que la detección de portadores, conjuntamente con la implementación de medidas de descolonización, es efectiva para disminuir las tasas de infección del sitio operatorio asociadas con intervenciones cardiovasculares, así como una estrategia alternativa para la contención de posibles brotes causados por este microorganismo.
